# Estimating the Climate Niche of *Sclerotinia sclerotiorum* Using Maximum Entropy Modeling

**DOI:** 10.3390/jof9090892

**Published:** 2023-08-31

**Authors:** Susan D. Cohen

**Affiliations:** Center for Regulatory Research, LLC, 2355 Highway 36 West, Suite #400, Minnesota, MN 55113, USA; sdcohen50@gmail.com; Tel.: +1-651-251-6965

**Keywords:** *Sclerotinia sclerotiorum*, maximum entropy modeling, climate niche, bioclimatic variables

## Abstract

*Sclerotinia sclerotiorum*, a fungal pathogen, causes world-wide crop losses and additional disease management strategies are needed. Modeling the climate niche of this fungus may offer a tool for the selection of biological control organisms and cultural methods of control. Maxent, a modeling technique, was used to characterize the climate niche for the fungus. The technique requires disease occurrence data, bioclimatic data layers, and geospatial analysis. A cross-correlation was performed with ArcGIS 10.8.1, to reduce nineteen bioclimatic variables (WorldClim) to nine variables. The model results were evaluated by AUC (area under the curve). A final model was created with the random seed procedure of Maxent and gave an average AUC of 0.935 with an AUC difference of −0.008. The most critical variables included annual precipitation (importance: 14.1%) with a range of 450 mm to 2500 mm and the mean temperature of the coldest quarter (importance: 55.6%) with a range of −16 °C to 24 °C, which contributed the most to the final model. A habitat suitability map was generated in ArcGIS 10.8.1 from the final Maxent model. The final model was validated by comparing results with another occurrence dataset. A Z-Score statistical test confirmed no significant differences between the two datasets for all suitability areas.

## 1. Introduction

*Sclerotinia sclerotiorum* (Lib.) de Bary, an ascomycetous fungal plant pathogen, is found worldwide on over 500 plant species such as sunflower, soybean, oilseed crops, peanut, onion, and tulips within dicot and monocot plant groups [1,2,3,4]. This fungal disease was previously reported to cause over USD 200 million in annual losses in the USA [2,4], and soybean production reports (2010 to 2014) indicate 2.8 million metric tons of yield loss with a loss value of USD 1.2 billion [2,5,6,7]. Disease reports indicate that *S. sclerotiorum* is found in most parts of the world, including Canada, the USA, Central America, South America, Africa, Europe, Asia, and Oceania, and the disease it causes is commonly known as Sclerotinia stem rot or white mold disease depending on the infected host plant species [3,8,9,10]. *S. sclerotiorum* produces long-term survival structures called sclerotia, which develop in diseased plant tissue and then fall to the ground and enter the soil [11,12,13]. The sclerotia germinate, giving rise to apothecia and ascospores. The ascospores germinate and infect plant hosts initiating the disease symptoms. This disease develops under cool, moist conditions and is widespread [3,12]. In the Mediterranean area, little or no disease occurs in the summer [12]. The disease is difficult to manage and focusing on the fungal responses to environmental conditions worldwide may give another tool to compare chemical and biological treatments. Using a preventative and ongoing disease management approach by identifying potential geographic habitats and specific environmental conditions via a modeling technique now and under climate change in the future would allow for early application and monitoring of disease management tools in specific locations. One particular modeling approach, species distribution modeling using maximum entropy modeling (Maxent [14,15,16]), may offer prevention and ongoing monitoring tools for disease management based on collection of disease occurrence records.

Using maximum entropy modeling (Maxent), the prediction of species’ geographic distributions has been successfully carried out with occurrence data from museums and herbariums [17]. Maxent has been used to define species’ geographic distributions by determining the environmental requirements within the limits of those environmental variables [15]. Maxent is categorized as a machine learning method, builds multiple models iteratively, and works with presence-only species data and presence-absence species data [14,16]. This technique has been used in several fungal studies to predict potential geographic distribution for conservation management of medically important fungi such as *Aureoboletus projectellus*, *Ganoderma lucidum*, and Sanghuang (*Phellinus baumii*, *P. igniarius*, *P. vaninii* species) [18,19,20]. In addition, studies conducted with *Austropuccina psidii* examined fungal and susceptible/resistant myrtle hosts under climate change conditions as a strategy for plant host conservation and disease management [21]. Maximum entropy modeling can also be applied to plant disease management and habitat suitability with the fungal pathogen, *S. sclerotiorum*, using presence-only data from herbarium and culture collections. This study will use Maxent modeling to predict geographic distributions based on climate variables, determine the climate niche of the fungus, and assess worldwide geographic areas suitable for the growth of the fungal pathogen, *S. sclerotiorum*. Once determined, the climate niche of *S. sclerotiorum* can be compared with the climate niches of current and potential biological control organisms [22].

## 2. Materials and Methods

### 2.1. Species Occurrence Data

**Figure 1:**
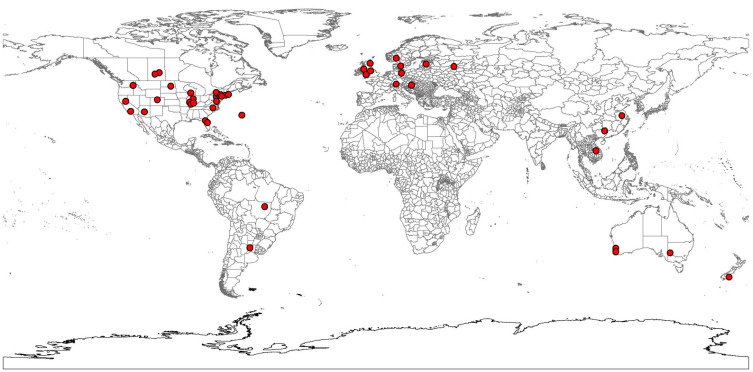
Spatially Thinned Occurrence Data of the Plant Pathogen, *Sclerotinia sclerotiorum*, used in Maxent Analysis. Red dots represent the worldwide occurrence locations of the plant pathogen.

The Mycological Portal to fungal herbarium and culture collections (http://mycoportal.org/portal/index.php, accessed on 25 August 2021) provided geographic distribution data for *S. sclerotiorum*, for Maxent bioclimatic modeling studies (Table S1, Mycological Portal, 67 records, 17 countries). A world validation dataset was generated separately from the Global Biodiversity Information Facility (GBIF.org (11 May 2021) GBIF Occurrence Download https://doi.org/10.15468/dl.5euyph) and the scientific literature [23] without the occurrence data points used in the initial Maxent model analysis (Table S2, GBIF, 85 records, 13 countries). A spreadsheet file for *S. sclerotinia* was created in Microsoft Office Excel 365 containing columns of distribution data with the fungal species name, latitude, longitude, and locality names and then exported to a CSV text file. The text file was added as a layer to ArcGIS Desktop ver. 10.8.1 geographic information system software (ESRI, Redlands, CA, USA) containing world map layer from GADM ver. 3.4 April 2018 (https://gadm.org/, accessed on 10 December 2021). For example, location coordinates were adjusted if the data specified a land area but appeared as an ocean location instead. The clustering of occurrence points leading to spatial bias was removed through spatial thinning using the Wallace module in R [24]. The initial dataset (Table S1) containing 67 occurrences was reduced to 45 by removing those points within 100 km of each other (Table S3) (Figure 1).

### 2.2. Taxonomy and Life Cycle of Sclerotinia sclerotiorum

*S. sclerotiorum* taxonomy follows the classification of kingdom Fungi, phylum Ascomycota, class Leotiomycetes, order Helotiales, family Sclerotiniaceae, genus *Sclerotinia*, and species *sclerotiorum* [25]. During the life cycle of the fungus, *S. sclerotiorum* produces survival sclerotia structures which can either germinate myceliogenically in the presence of nutrients or germinate carpogenically after environmental conditioning [2,4,26]. Plant infection can occur by myceliogenic germination from sclerotia in soil or by ascospores released from apothecia developed from the carpogenic germination of sclerotia [2,26]. Apothecia release ascospores which are carried by wind currents and land on nearby susceptible plant hosts [4]. Ascospores require water to germinate and nutrients to infect healthy plant tissue [12,27]. Sclerotia are known to survive for up to 8 years in soil [11,13].

### 2.3. Climate Data and Environmental Variables

The average of nineteen bioclimatic environmental variables for the years 1970–2000, version 2.1, and at the 2.5 min level were downloaded from the World Climate Organization (https://www.worldclim.org/data/worldclim21.html, accessed on 10 April 2020) and are listed in Table 1.

**Figure 2:**
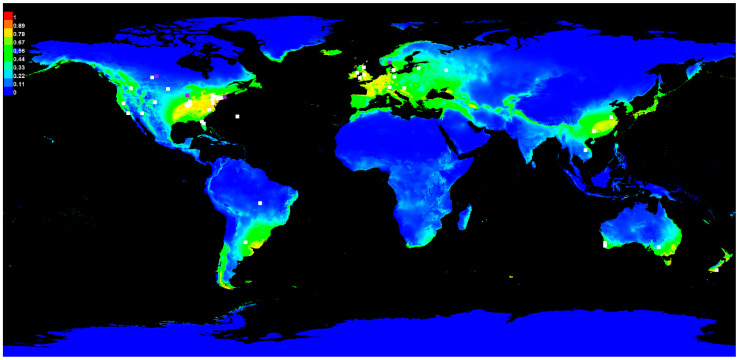
Final Maxent Model 7 of plant pathogen, *Sclerotinia sclerotiorum*. White symbols are training locations and purple symbols are test locations. Red indicates a high probability of suitable conditions for occurrence, green indicates typical conditions for occurrence, and lighter shades of blue indicate low predicted probability of suitable conditions on the color legend in the upper left of the figure.

Data from nineteen variables listed in Table 1 were downloaded from the WorldClim website, extracted as Geotiff files, imported into ArcGIS Desktop ver. 10.8.1 (https://www.esri.com/, accessed on 12 December 2018), and converted to ASCII format (asc) using the conversion tool in the ArcToolbox 10.8.1. A cross-correlation between bioclimatic variables was conducted with the band collection statistics tool in the ArcToolbox and bioclimatic variables containing related data were removed before conducting the Maxent analysis (Table S4). Nineteen variables were reduced to nine variables (Table 1). Before selecting the bioclimatic variables, all variables were included in a Maxent baseline model run to determine the initial contribution percentage of each variable. If a correlation between two variables was greater than 0.8, the most relevant variable based on contribution to the model is retained and the other variable is removed from consideration. The remaining variables were included if the percentage of contribution was greater than 1.0 (Table S4) [28,29]. The selected nine variables highlighted in Table 1 were then included in the final random seed Maxent model run to identify the top two bioclimatic variables for the climate niche evaluation (Figure 2).

### 2.4. Maxent Model Considerations

Maxent ver. 3.4.1 modeling software [15,16] was downloaded from the web at https://biodiversityinformatics.amnh.org/open_source/maxent/ (accessed on 12 December 2018) and installed under the Windows 10 operating system. The software requires the import of data occurrence files containing geographic coordinates and bioclimatic variables before conducting the modeling analysis [14,15,16]. The file format included three data columns (the scientific name, the digital longitude, and the latitude coordinates) and was saved as a CSV text file. Once the occurrence files were created, the files were imported into the Maxent software. The bioclimatic variables converted previously in ArcGIS Desktop ver. 10.8.1 from GeoTiff format to ASCII (asc) format were also imported into Maxent. Model parameters included presence data, random 25% training data, (β) regularization of 1, the maximum number of background points of 10,000, auto features, and logistic output with and without the jackknife process. The default for background points was selected based on matching the geographic extent and inclusion of diverse habitat areas for the presence-only Maxent model [30,31]. The use of a small spatially thinned sample size with large geographic extent is well handled by Maxent [32]. Maxent software also outputs graphic models, graphs, and spreadsheet tables of occurrence predictions (Figure S1). The impact of bioclimatic variables on fungal occurrences can be further evaluated by examining the data from the jackknife process. The jackknife process reports on the % of contribution and permutation importance each bioclimatic variable has on the model (Figure S2). Prediction measurements for models are based on the area under the receiver operating characteristic curve (AUC) on a graph. An AUC value of 0 indicates that 0% of occurrences were predicted correctly and an AUC value of 1 indicates that 100% of the predictions were correct. To select the best Maxent model for *S. sclerotiorum*, the same fungal occurrence data and 9 bioclimatic variables were again run using the random seed and the same selected parameters as before, thereby generating 10 random models. The best model for *S. sclerotiorum* was then selected based on the AUC difference value with the minimum difference from the AUC training–AUC test results [33]. The AUC measure was used instead of an alternative such as TSS (True Skill Statistic, sensitivity+specificity-1) [34] which is based on specificity (proportion of absences correctly predicted). In a presence-only Maxent model, the background points are not equivalent to absences [31].

Additional parameters for optimizing the Maxent model include the regularization multiplier (β) and the feature combination which may affect the predictive performance of Maxent modeling. Optimizing these parameters may reduce the overfitting of the output and lead to better results when applying the model’s use to novel environments [30]. The (β) multiplier tends to affect the focus of the output. For example, a smaller (β) will fit the occurrence data more closely, leading to overfitting. The larger (β) will produce a prediction that can be used in a wider area. In this case, to select the best fit, (β) = 1 was used to apply to the entire world landscape. The other parameter to consider is the feature combination derived from the climate bio-variables. These features are linear (L), quadratic (Q), product (P), threshold (T), and hinge (H). The number of datapoint occurrences dictates the feature combinations to use in the model. For example, linear features are always used in the model, quadratic features are used when the number of occurrences is more than 10, hinge features are used with more than 15, and threshold and product features are used when the occurrences are more than 80 [30]. The feature type used in this study was HLQ (hinge, linear, quadratic).

### 2.5. Variable Selection and Climate Niche

Selection of bioclimatic variables for the final model random seed model climate niche is based on the percentage of contribution and permutation importance (Table 1) using the jackknife analysis procedure in the Maxent software. Philipps [35] describes the percentage of contribution as a way to keep track of which environmental variables contribute to the fitting of the model. Each step of the Maxent algorithm increases the gain of the model by modifying the coefficient for a single feature and the program assigns the increase in the gain to the environmental variable that the feature depends on. This value depends on the particular Maxent codes used to obtain the optimal solution and a different algorithm could obtain the same solution via a different path resulting in a different percent contribution. Also, if any correlated environmental variables are used, the value of the percent contribution would change. Percent contribution should be viewed with caution. Therefore, the second selection feature, permutation importance, depends only on the final Maxent model, not the path used to obtain it. The contribution for each variable is determined by randomly permuting the values of the variable among the training points (both presence and background) and measuring the resulting decrease in training AUC. A large decrease indicates that the model depends heavily on that variable. Values are normalized to give percentages. The jackknife analysis selected the environmental variable with the highest gain used in isolation as BIO11 (mean temperature of the coldest quarter). Also, BIO11 appears to have the most useful information by itself. The environmental variable that decreases the test gain the most when it is omitted is BIO12 (annual precipitation) which therefore appears to have the most information that is not present in the other variables (Figure S2).

**Figure 3:**
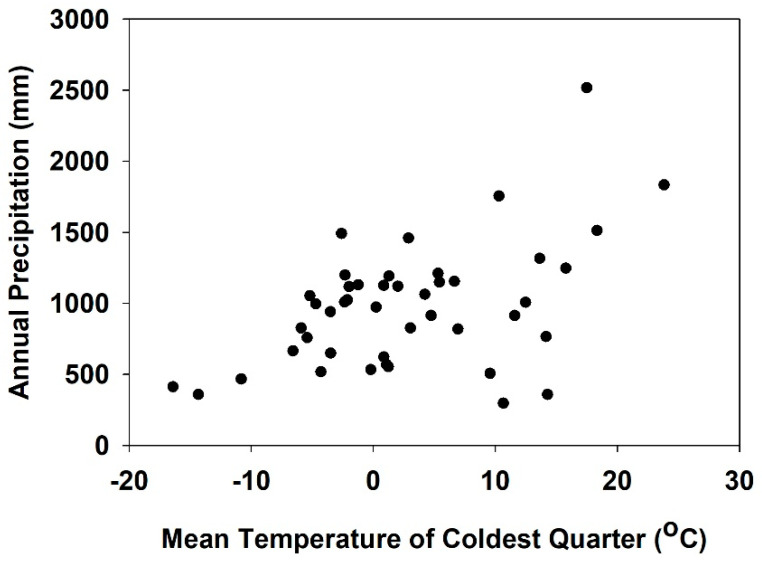
Climate niche of the plant pathogen, *S. sclerotiorum*, based on annual precipitation vs. mean temperature of the coldest quarter. Black dots represent annual precipitation and mean temperature of coldest quarter from Worldclim weather data (https://www.worldclim.org, accessed on 12 December 2018).

The climate niche for *S. sclerotiorum*, the fungal plant pathogen, was determined by selecting the bioclimatic variables (BIO11, BIO12) for the final random seed model 7 (Table 2) as described above and then extracting the values from the Maxent model as an asc file using the ArcToolbox to generate xy coordinates [20]. The BIO11 (mean temperature of coldest quarter) contributed 31.1% and 55.6% importance to the model. BIO12 (annual precipitation) contributed 14.2% and 14.1% importance to the model. BIO12 (annual precipitation) was selected over BIO19 (precipitation of coldest quarter) based on the performance in the jackknife analysis as BIO12 decreases the test gain the most when omitted and appears have the most information that is not present in other variables (Figure S2). The Maxent model for the climate niche (Figure 3) was further analyzed by a scatterplot using Sigmaplot ver. 12.5 (https://systatsoftware.com/, accessed on 12 December 2018).

### 2.6. Model Evaluation and Selection

Ten *S. sclerotiorum* models were generated from the random selection feature of Maxent with 9 climate biovariables. These biovariables included BIO3 (isothermality), BIO4 (temperature seasonality), BIO5 (maximum temperature of warmest month), BIO6 (minimum temperature of coldest month), BIO11 (mean temperature of coldest quarter), BIO12 (annual precipitation), BIO14 (precipitation of driest month), BIO15 (precipitation seasonality), and BIO19 (precipitation of coldest quarter). The final model selection was based on the least difference between the AUC (area under the curve) training and AUC test values. The AUC values from the test dataset are considered more representative of the actual model than the training dataset. The AUC difference method was selected to minimize the risk of overfitting the model based only on the training dataset [33]. In addition, the Maxent modeling software evaluates the accuracy of the predictions by comparing the fractional value versus the cumulative threshold. For example, the omissions of training and test samples are compared to the predicted omission rate when using a particular model. The Maxent software also generates a sensitivity (1-omission rate) versus specificity (1-specificity or fractional predicted area) graph of training and test data for an individual model and then compares the results to the expected random prediction of AUC = 0.5 (Figure S1). The importance of the bioclimatic variables on the model is also considered using the jackknife analysis available in the Maxent software. The jackknife analysis evaluates the training gain, test gain, and AUC values without a bioclimatic variable, with one bioclimatic variable, and with all bioclimatic variables (Figure S2).

### 2.7. Fungal Species Habitat Suitability

The *S. sclerotiorum* model generated from the random seed model predictions (asc files) was added as a raster data layer overlaid onto a world map layer from GADM (https://gadm.org/, accessed on 10 December 2021) in ArcGIS Desktop ver. 10.8.1. The coordinate system of the map was defined as WGS 1984. The map was then reprojected to WGS 1984 Equal Earth Greenwich prior to the habitat suitability study. The reclassify tool in the spatial analyst extension of the ArcGIS Desktop toolbox was used to classify the areas into equal intervals, update the range of values in the attribute table, and label the types of habitat suitability areas [36]. The ranges of habitat suitability included low suitability areas (0–0.2), moderate suitability areas (>0.2–0.4), high suitability areas (>0.4–0.6), and very high suitability areas (>0.6–1.0). An additional worldwide dataset was created from the Global Biodiversity Information Facility (Copenhagen, Denmark, https://www.gbif.org/, accessed on 10 December 2021) without locations from the spatially thinned *Sclerotinia* Maxent analysis in EXCEL to test the consistency and validity of the model. The dataset was added as a layer to the habitat suitability map. Habitat suitability areas were determined for each data point. Occurrence numbers for each suitability area were selected as an attribute search in ArcGIS desktop 10.8.1 for both the Maxent model dataset and the validation dataset (Table 3). A statistical comparison of differences between the Maxent model dataset and the validation dataset was carried out with SigmaPlot 12.5 software (https://systatsoftware.com/, accessed on 10 December 2021) using the comparison of the proportions of two populations (primary Maxent model and valid occurrence datasets) using the Z-Score at the *p* = 0.05 level to determine if the populations are equal or different for the four suitability areas.

## 3. Results

### 3.1. Sclerotinia Model Prediction

Random model 7 (Figure 2) was selected for further analysis of accuracy, sensitivity, and specificity as shown (Figure S1). Training and test samples were compared to predicted omission and results favored the slope of predicted omission (Figure S1). Sensitivity vs. specificity of training (AUC = 0.931) and test data (AUC = 0.939) were also examined and were significantly higher than the random prediction of AUC = 0.5 (Figure S1). A comparison of the environmental variables’ impact on the model was measured by the Maxent jackknife analysis (Figure S2). Bioclimatic variable BIO11 had the highest gain and most impact on the training and test datasets. Bioclimatic variable BIO6 had the second highest impact on the training dataset, while bioclimatic variable BIO12 had the second highest impact on the test dataset. Bioclimatic variable BIO12 was considered important as it contained the most information that was not present in other variables for the training dataset. Similar results occurred with the test dataset (Figure S2). Comparison of AUC ratings under jackknife analysis were not as clear-cut, possibly due to combining training and test datasets. Based on contribution and importance, bioclimatic variables BIO11 and BIO12 were considered a good fit for the model and also contained the most information that was missing from the other climate variables.

The AUC or area under the curve (Table 2) is used as a measure of the accuracy of each Maxent model. The initial model results based on 45 occurrences with 34 in the training dataset and 11 in the test dataset generated an average AUC of 0.920. This is considered a very reliable model prediction. The final Maxent model was generated by the random seed feature of Maxent by randomly producing 10 potential models using the initial model bioclimatic variables and parameters. In Table 2, the final random model 7 for *S. sclerotiorum* was selected based on the AUC difference of −0.008. Figure 2 represents the Maxent analysis illustrating the training and test data points of the final Maxent model 7 of *S. sclerotiorum*.

### 3.2. Climate Responses and Climate Niches

Based on the Maxent random seed models (Table 1), the two highest contributing variables included BIO11 (mean temperature of the coldest quarter) at percentage = 31.1% and permutation importance = 55.6%) and BIO12 (the annual precipitation) at percentage = 14.2% and permutation importance = 14.1% for the *S. sclerotinia* random seed model. The climate niche for *S. sclerotiorum* was further defined by extracting the bioclimatic data from each of the two highest contributing variables, thereby generating xy coordinates for the scatter plot. Based on the scatterplot, the climate niche for *S. sclerotiorum* was defined by annual precipitation vs. the mean temperature of the coldest quarter (Figure 3). Results for *S. sclerotiorum* using the mean temperature of the coldest quarter (BIO11) were in the range of −16 °C to 24 °C with annual precipitation (BIO12) from 450 mm to 2500 mm (Figure 3).

### 3.3. Geographic Habitat Suitability and Model Validation

**Figure 4:**
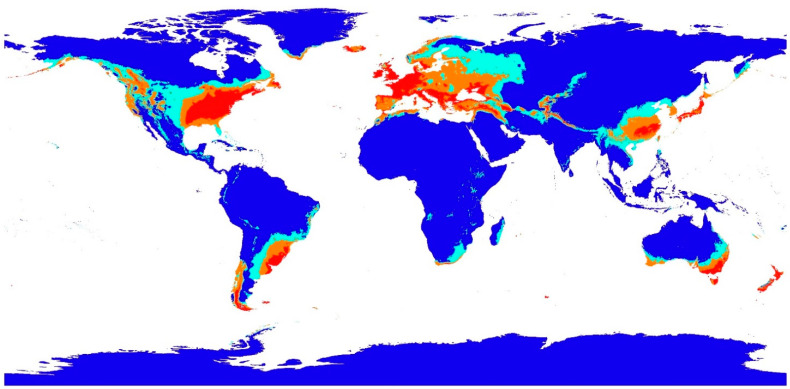
*S. sclerotiorum* habitat suitability map reclassified from Final Maxent Model 7. Blue color represents low suitability areas (0–0.2), turquoise color represents moderate habitat suitability (>0.2–0.4), tan color represents high habitat suitability (>0.4–0.6), and red color indicates very high habitat suitability (>0.6–1.0). The map shows the results of the Maxent model, but other environmental factors could alter the presence of *S. sclerotiorum*.

**Figure 5:**
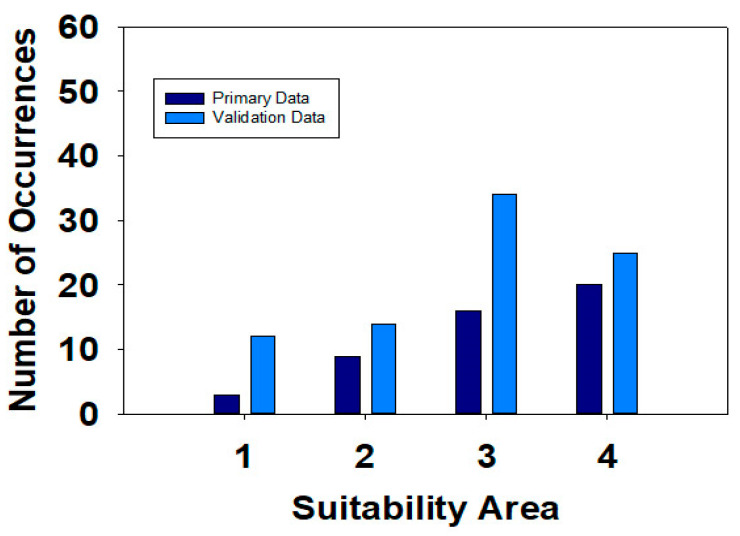
Comparison of the number of occurrences between the primary (Maxent model dataset) and the validation model dataset based on suitability region areas. Suitability areas include 1 = low suitability, 2 = moderate suitability, 3 = high suitability, and 4 = very high suitability.

*S. sclerotiorum* habitat suitability areas are defined as low suitability areas (0–0.2), moderate suitability areas (>0.2–0.4), high suitability areas (>0.4–0.6), and very high suitability areas (>0.6–1.0) (Figure 4). *S. sclerotiorum* occurrences (Table 3) were overlaid onto the final Maxent suitability model in ArcGIS desktop 10.8.1 to evaluate the number of occurrences in each suitability area. The habitat suitability regions in the Maxent model dataset included 3 occurrences in the low suitability area, 5 occurrences in the moderate area, 16 occurrences in the high area, and 20 occurrences in the very high area. Of the total number of 45 occurrences, the majority of occurrences (93.3%) were classified in moderate to very high suitability areas. The validation dataset contained 85 occurrences with 12 in the low suitability area, 14 in the moderate suitability area, 34 in the high suitability area, and 25 in the very high suitability area. The majority of the occurrences (85.9%) in the validation dataset covered the moderate to very high suitability areas (Figure 5). A comparison of the two datasets by suitability categories was analyzed by a Z-Score test of two population proportions at the 0.05 probability level. The Z-Score test indicated no significant differences between the primary (Maxent model dataset) and the validation dataset at *p* = 0.05 for all four suitability areas. Under this statistical test, the two datasets are similar, suggesting that the distribution, habitat suitability areas, and the Maxent model are representative of the climatic niche for *S. sclerotiorum* world distribution.

## 4. Discussion

Pearman et al. [37] describe the species niche as the requirements of a species to maintain a positive population growth rate. Earlier work by Hutchinson [38,39,40] defined two types of species niches such as a fundamental niche and a realized niche. The fundamental niche focuses on the requirements of a species necessary for positive population growth rates but disregards biotic interactions. The realized niche is the part of the fundamental niche with a positive growth rate, given the constraining effects of competition from biological interactions. Species’ growth rates can also be affected by environmental and climate interactions. Pearman et al. defines the environmental niche as all environmental conditions that meet the physiological requirements of a species necessary for positive population growth rates [37]. The climatic niche refers to an aspect of the environmental niche that is defined by limits in climate variation. Outside of this niche, a population cannot maintain a positive rate of growth during extreme winter or summer temperatures, excess precipitation, and drought. This study is focused on identifying a climate niche for the plant pathogen, *S. sclerotiorum*, using maximum entropy modeling. The results of this study have established a baseline climate niche for *S. sclerotiorum.* This climate niche ranges in mean temperature for the coldest quarter from −16 °C to 24 °C and annual precipitation from 45 mm to 2500 mm. The climate niche model predicts the geographic areas of suitability (moderate to very high) for *S. sclerotiorum* to include most of the USA, parts of Canada, Brazil, Uruguay, Argentina, Chile, the majority of European countries, parts of India, China, all of Japan, parts of Australia, and all of New Zealand.

Previous laboratory temperature and moisture studies limited testing conditions when examining results for sclerotial germination, so direct comparisons to this study are not appropriate, although comparison between laboratory and field studies has shown a close relationship [26]. However, laboratory studies with limited conditions do confirm sclerotia germination with temperatures of 10 °C and 15 °C. For example, Huang and Kozub’s study [41] confirm that the carpogenic germination of sclerotia of *S. sclerotiorum* is regulated by temperature and not by the geographic origin of the isolate. Fungal isolates from various geographic areas in the world were grown on potato dextrose agar in the laboratory at 10 °C and 25 °C. The sclerotia were harvested and placed on moist sand at 20 °C under light for 3 weeks to induce apothecia. Isolates from cool climates such as Hokkaido, Japan; North Dakota, USA; Alberta, Saskatchewan; and Manitoba, Canada, produced at 10 °C easily germinated but cultures from warm climates such as Taiwan, California, Florida, and Hawaii failed to germinate. Warm climate isolates required an additional four weeks of 10 °C conditioning under moist conditions to promote germination. In addition, the plant host origin had no bearing on the germination response as isolates were derived from a range of plant hosts. Hao et al. [42] further defined temperature and moisture conditions for the carpogenic germination of *S. sclerotiorum* based on conditioning sclerotia in the soil as 15 °C and −0.03 or −0.07 MPa. Mycelial gemination rarely occurred under experimental conditions. Soil type did not affect myceliogenic or carpogenic germination of sclerotia. Apothecia production factors of light, temperature, and moisture were further defined by Sun and Yang [43]. At low light intensity (80 to 90 mol m^−2^ s^−1^), optimal temperatures ranged from 12 °C to 18 °C irrespective of soil moisture. With high light intensity (120–130 mol m^−2^ s^−1^), the optimal temperature was 20 °C with high soil moisture. Apothecial initials were slow to develop and appeared thin under the low light intensity. Light intensity and soil types were not included in this study. Further studies using maximum entropy modeling would be needed to refine the climate niche to include apothecia production and ascospore release, differences between cultures from temperate and warm climates, and niche changes under climate stress.

Examination of past field studies has confirmed the climate niche results of this study. Schwartz and Steadman [44] investigated the population dynamics of the fungus under field conditions. Results indicated that when low numbers of sclerotia were present followed by irrigation conditions, sclerotia gave rise to apothecia with repeated cycles of high numbers of ascospores. Weiss et al. [45] conducted temperature/moisture studies in a growth chamber and monitored temperature and moisture from irrigation in field studies after inoculation. In growth chamber studies, disease developed between 10 °C and 25 °C with 100% humidity. The disease did not develop at 30 °C. Field trials conducted in Western Nebraska compared two irrigation conditions (normal = 5.5 cm of water per 10 days and heavy irrigation = 5.5 cm of water every 5 days). Air temperatures ranged from 10 °C to 30 °C. Results of the field experiments indicated that only 3% of the plants were diseased in the normal irrigation treatment compared to 40% of the plants in the heavy irrigation treatment. Authors suggest that the duration of leaf moisture, not air temperature, limited the disease development. Vegetable field trials conducted by Moore [46] in south Florida also came to similar conclusions. Many of the conditions described by these authors that impact disease severity are not covered in the present model. *Sclerotinia* disease conditions for temperature and moisture were monitored for over 10 years (1944–1954) [46]. Mean temperatures, rainfall amounts, and days of rain were recorded at two different location sites, Homestead and Pompano Beach, during the winter months of December, January, and February. Disease severity was rated light or moderate to severe. Average temperatures for Homestead were recorded from 19.1 °C to 21.8 °C (light disease) and 17.2 °C to 21.8 °C (moderate to severe disease) and average temperatures for Pompano Beach were recorded as 19.3 °C–21.8 °C (moderate to severe disease). Temperatures recorded at both locations were not considered a factor in the disease progression. Average rainfall and number of rain days for Homestead (light disease = average rainfall 20.8 mm–32 mm, 4.2 days–7.7 days; moderate to severe disease = average rainfall for 35.6 mm–45.4 mm, 5.1 days–6.5 days) as well as for Pompano Beach (light disease = average rainfall 22.4 mm–58.4 mm, 3.0 days–5.8 days; moderate to severe disease = average rainfall 44.5 mm- 69.9 mm, 5.8 days–6.0 days) had the most impact on disease progress. The severity of the disease was most affected by the amount of rain available during the winter months. These specific conclusions will vary depending on the growing season in a particular cropping location. Disease incited by *S. sclerotiorum* was most severe at the Homestead location and the author attributed that result to the fact that this location had a marl soil type that holds moisture whereas the Pompano Beach location had a sandy soil type with better drainage.

In this study, climate responses of temperature and moisture were modeled from *S. sclerotiorum* geographic occurrences and thereby predicted potential fungal coverage worldwide. Based on this method, the results allowed for the development of a climate niche for the fungus related to the precipitation of the driest month vs. the mean temperature of the coldest quarter. The mean temperature ranged from −16 °C to 24 °C and annual precipitation from 45 mm to 2500 mm. In summary, the fungus is quite tolerant of low temperatures but not high temperatures and prefers moisture but tolerates dry conditions. These results confirm studies discussed earlier in the symposium paper on *S. sclerotiorum* [27]. In the future, further research studies for this project should include determining the climate niche for sclerotia and apothecia, using samples identified to the species level by genomic analysis, life stage analysis using transcriptomic and metabolomic datasets, sample size effects, moisture with soil effects on climate, and estimating geographic extent. Additional worldwide studies with fungal pathogens, saprophytes, and mycorrhizae [47,48,49] suggest that climate is also a driving force for the prediction of the growth and distribution of fungi. Vetrovsky et al. [49] completed a meta-analysis of fungal studies and determined that fungal plant pathogens have a broader climate niche than other fungal groups. Also, climate (temperature and precipitation) contributed a larger impact on plant pathogen distribution than soil and vegetation [49].

Using maximum entropy and habitat suitability methods, it is possible to develop a worldwide geographic bioclimatic model for *S. sclerotiorum*. Potential biological control organism models could be screened for similarities to the pathogen model [22]. Matching the pathogen and biological control organism based on the closest climate niche fit might lead to a better selection protocol for disease control. In addition, examining the climate niche under climate stresses will offer a better perspective of the climate impacts on the pathogenicity of the pathogen [50,51] as well as the potential effectiveness of biological control agents. Also, examining environmental conditions geographically has been effective at predicting new locations suitable for invasive plant diseases [52] and potential areas suitable for endangered species [53,54]. As with all modeling projects, there are some caveats to be considered. For monitoring projects, the geographic extent is important in order to cover multiple habitat and topography changes. Small-scale and large-scale projects may miss changes in geography. A survey of the geography prior to starting the project is worthwhile in order to determine the geographic extent of the model. Fungal identification is important for samples that are used in the modeling project. Fungal databases should not only include correct geographic coordinates but also precise species identification by morphology and molecular means [55]. Improved identification of samples will ensure more uniformity of samples and indicate new genetic strains that may perform differently in the environment. In addition, models need to be validated by compiling and running a separate independent sample data set [56].

## Figures and Tables

**Table 1:** Percentage contribution and permutation importance of bioclimatic variables used in maximum entropy analysis (Maxent) for *S. sclerotiorum* random seed model 7.

Bioclimatic Variable *	Unit	Percentage	Permutation
		Contribution	Importance
BIO1 = Annual Mean Temperature	°C	__	__
BIO2 = Mean Diurnal Range (Mean of monthly (max temp − min temp))	°C	__	__
BIO3 = Isothermality (BIO2/BIO7) (* 100)	**__**	**8.5**	**11.9**
BIO4 = Temperature Seasonality (standard deviation * 100)	**C of V**	**0.5**	**6.4**
**BIO5 = Max Temperature of Warmest Month**	**°C**	**2.6**	**2.9**
**BIO6 = Min Temperature of Coldest Month**	**°C**	**0.4**	**0.0**
BIO7 = Temperature Annual Range (BIO5–BIO6)	°C	__	___
BIO8 = Mean Temperature of Wettest Quarter	°C	__	___
BIO9 = Mean Temperature of Driest Quarter	°C	__	___
BIO10 = Mean Temperature of Warmest Quarter	°C	__	___
**BIO11 = Mean Temperature of Coldest Quarter**	**°C**	**31.1**	**55.6**
**BIO12 = Annual Precipitation**	**mm**	**14.2**	**14.1**
BIO13 = Precipitation of Wettest Month	mm	__	__
**BIO14 = Precipitation of Driest Month**	**mm**	**11.5**	**1.6**
**BIO15 = Precipitation Seasonality (Coefficient of Variation)**	**C of V**	**7.0**	**0.3**
BIO16 = Precipitation of Wettest Quarter	mm	__	___
BIO17 = Precipitation of Driest Quarter	mm	__	___
BIO18 = Precipitation of Warmest Quarter	mm	__	___
**BIO19 = Precipitation of Coldest Quarter**	**mm**	**24.2**	**7.1**

* The highlighted variables were selected with the ArcGIS Desktop ver. 10.8.1 band collection statistics using cross-correlation for modeling with Maxent 3.4.1 software. The percentage contribution and permutation importance of bioclimatic variables was generated by the jackknife analysis in Maxent software from the final random seed model 7 for *S. sclerotiorum*.

**Table 2:** Maxent analysis with nine bioclimatic variables using the Maxent random seed procedure *.

Species	Training #	Test #	AUC Training	AUC Test	AUC Difference	AUC Average
Fungal pathogen						
*S. sclerotiorum*						
Non-Random Model	34	11	0.936	0.903	0.033	0.920
Random Model 0	40	5	0.934	0.917	0.017	0.926
Random Model 1	40	5	0.937	0.875	0.062	0.906
Random Model 2	40	5	0.935	0.879	0.056	0.907
Random Model 3	40	5	0.930	0.970	−0.04	0.950
Random Model 4	40	5	0.929	0.955	−0.02	0.942
Random Model 5	41	4	0.929	0.941	−0.012	0.935
Random Model 6	41	4	0.937	0.861	0.076	0.900
Random Model 7	41	4	0.931	0.939	−0.008	0.935
Random Model 8	41	4	0.941	0.831	0.110	0.886
Random Model 9	41	4	0.936	0.842	0.094	0.889

* AUC = is a measure of model prediction accuracy based on the area underneath a curve. A model with AUC = 0 demonstrates 0% correct prediction whereas a model with AUC = 1 would have 100% correct prediction. The number of occurrences (#) used in the Training and Test groups.

**Table 3:** Habitat suitability area preference as determined by the S. sclerotiorum Maxent model.

Suitability Categories *	Number of Occurrences		Z-Score **	*p* Value **
	Maxent Model Dataset	Validation Dataset		
Low Suitability	3	12	−0.624	0.533
Moderate Suitability	9	14	−0.342	0.732
High Suitability	13	34	−0.369	0.712
Very High Suitability	20	25	0.731	0.465

* Original dataset of 45 total occurrences used for Maxent model. The validation dataset contained 85 occurrences to test for consistency of the *Sclerotinia* model. Suitability areas are classified as low suitability (0–0.2), moderate suitability (>0.2–0.4), high suitability (>0.4–0.6), and very high suitability (>0.6–1.0) as described in Section 2.7. ** Both datasets were determined not to be significantly different from each other by the Z-Score for two population proportion distributions with the *p*-value at the 0.05 level.

## Data Availability

Data is contained within the article or Supplementary Materials.

## Supplementary Materials

The following supporting information can be downloaded at: https://www.mdpi.com/article/10.3390/jof9090892/s1. Table S1: *S. sclerotiorum* occurrence dataset. Table S2: *S. sclerotiorum* world validation occurrence dataset. Table S3: *S. sclerotiorum* spatially thinned dataset for Maxent analysis. Table S4: *S. sclerotiorum* bioclimatic variables cross-correlation matrix table. Figure S1: Maxent random model 7: prediction accuracy, sensitivity, and specificity. Figure S2. Maxent jackknife analysis of environmental variables.
