# Supplementary material for: Estimating the Climate Niche of Sclerotinia sclerotiorum Using Maximum Entropy Modeling
Source: J Fungi (Basel). 2023 Aug 31;9(9):892. doi: 10.3390/jof9090892 (PMC10532795; doi:10.3390/jof9090892)

Cohen, SD. 2023. Estimating the climate niche of *Sclerotinia sclerotiorum* using maximum entropy modeling

Figure S1. Maxent Random Model: Prediction Accuracy, Sensitivity and Specificity

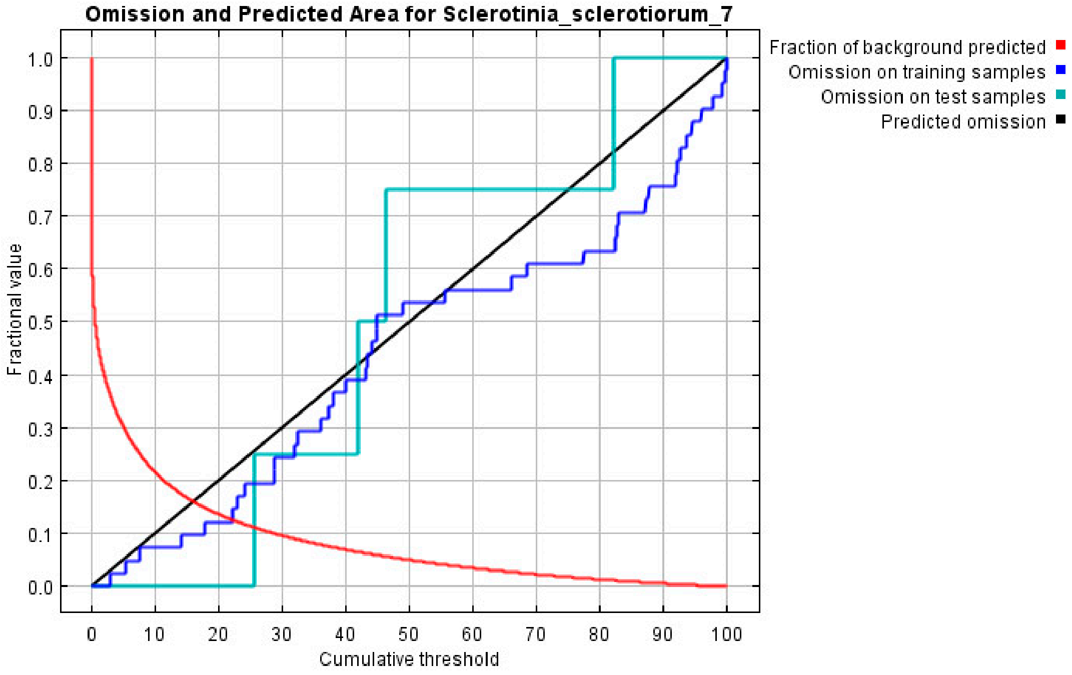

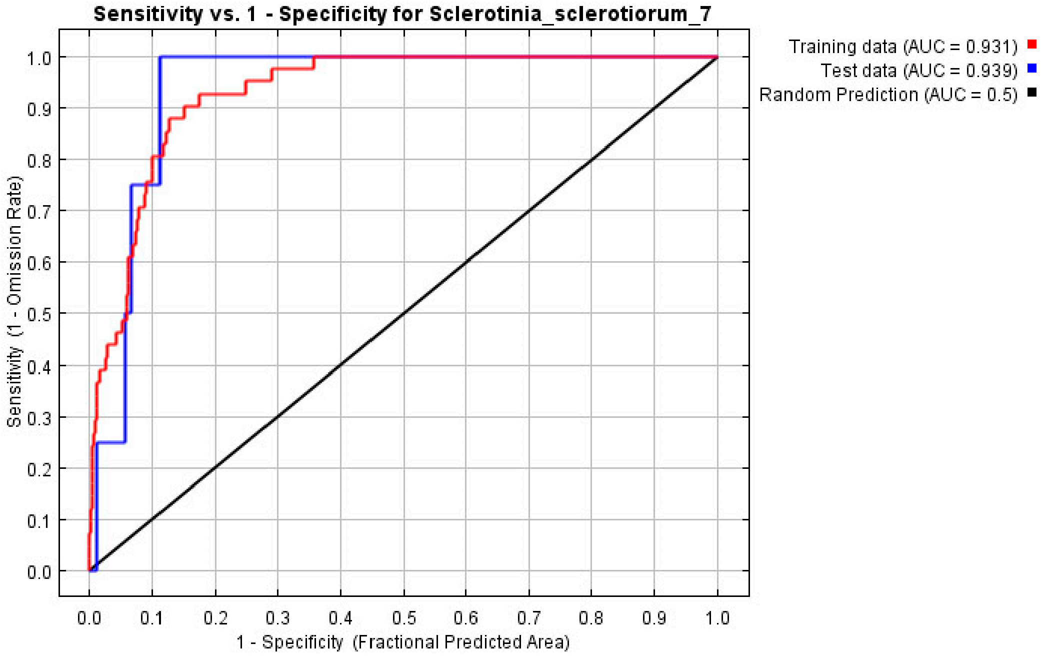

Supplement: Supplementary file 1 [file jof-09-00892-s001.zip › Figure S1 Maxent Random Model_ Predicted Accuracy, Sensitivity and Specificity 962023.pdf]
