# Supplementary material for: Estimating the Climate Niche of Sclerotinia sclerotiorum Using Maximum Entropy Modeling
Source: J Fungi (Basel). 2023 Aug 31;9(9):892. doi: 10.3390/jof9090892 (PMC10532795; doi:10.3390/jof9090892)
Supplement: Supplementary file 1 [file jof-09-00892-s001.zip › Figure S2 Maxent Jackknife Analysis of Environmental Variables 962023.pdf]

Cohen, SD. 2023. Estimating the climate niche of *Sclerotinia sclerotiorum* using maximum entropy modeling

Figure S2. Maxent Jackknife Analysis of Environmental Variables

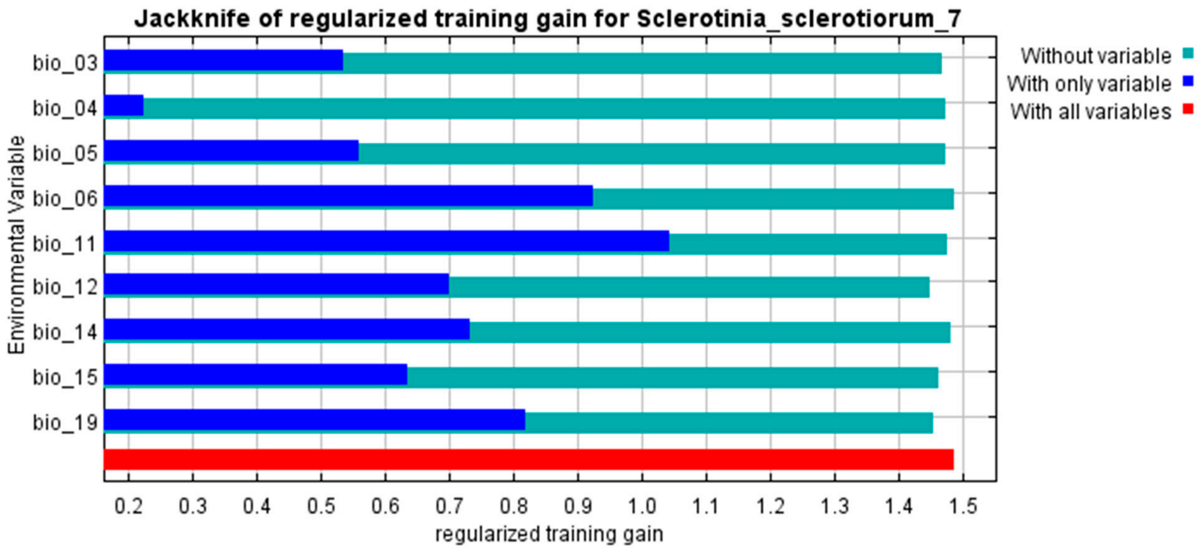

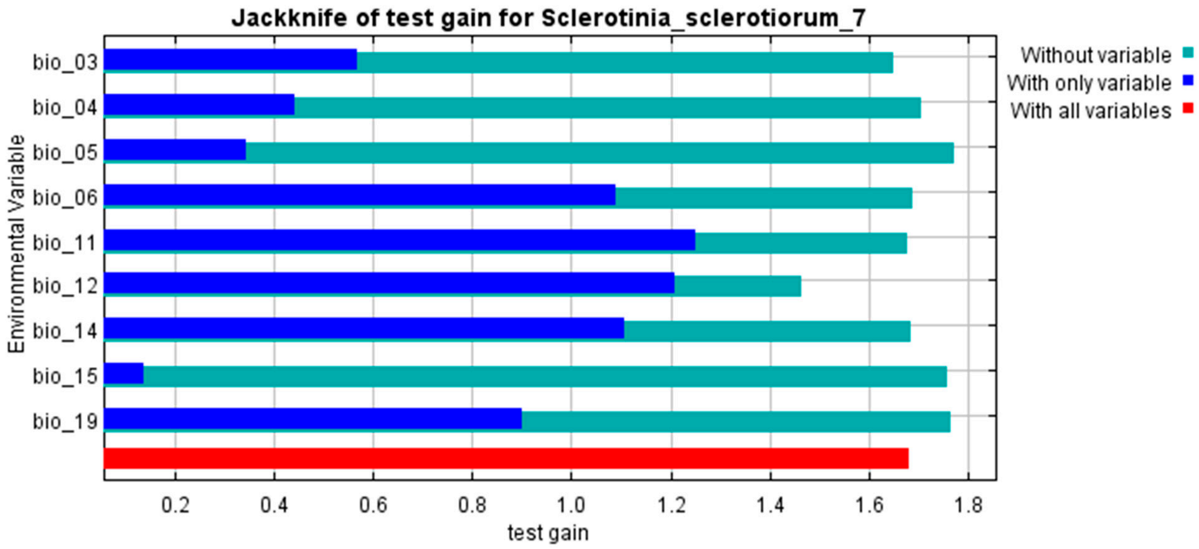

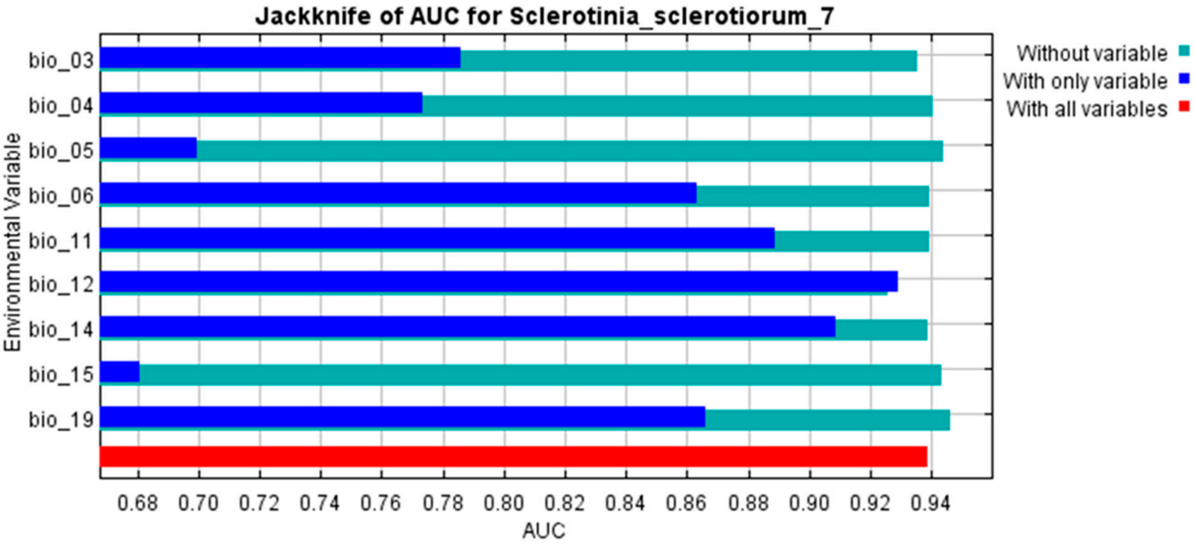

Jackknife of AUC for Sclerotinia_sclerotiorum_7
Without variable
With only variable
With all variables
Environmental Variable
bio_03
bio_04
bio_05
bio_06
bio_11
bio_12
bio_14
bio_15
bio_19
0.68
0.70
0.72
0.74
0.76
0.78
0.80
0.82
0.84
0.86
0.88
0.90
0.92
0.94
AUC
